# ﻿A hitherto overlooked article by Gressitt in 1941 (Insecta, Coleoptera, Cerambycidae, Lamiinae)

**DOI:** 10.3897/zookeys.1168.107021

**Published:** 2023-06-29

**Authors:** Mei-Ying Lin, Steven W. Lingafelter

**Affiliations:** 1 Engineering Research Center for Forest and Grassland Disaster Prevention and Reduction, Mianyang Normal University, 166 Mianxing West Road, Mianyang, Sichuan 621000, China Mianyang Normal University Mianyang China; 2 8920 South Bryerly Court, Hereford, Arizona, 85615, USA Unaffiliated Hereford United States of America

**Keywords:** Date of publication, homonym, nomenclature, synonym, West China Border Research Society

## Abstract

An article published by Gressitt (1941) has been ignored by all longicornists, including Gressitt himself. However, according to ICZN, it meets all criteria as an official publication, and this status affects three taxa that were formally described therein: Bacchisa (Bacchisa) rigida (Gressitt, 1941) = *Chreonomarigida* Gressitt, 1941 = *Chreonomarigida* Gressitt, 1942 homonym and **syn. nov.**; *Tetraophthalmussikang* (Gressitt, 1941) = *Chreonomasikang* Gressitt, 1941 = *Chreonomasikanga* Gressitt, 1942, **syn. nov.**; *Anastathesparvahainana* Gressitt, 1941 = *Anastathesparvahainana* Gressitt, 1942, homonym and **syn. nov.**

## ﻿Introduction

The first author changed her job from the Institute of Zoology, Chinese Academy of Sciences (Beijing, China) to Mianyang Normal University (Mianyang, Sichuan, China) in 2022. In her preparations to study the longhorned woodboring beetle (Coleoptera, Cerambycidae) fauna of Sichuan Province, Mr Yu-Tang Wang introduced the book series “Journal of the West China Border Research Society, Unabridged Photocopied Edition” to her. One article on the taxonomy of Cerambycidae written by J. Linsley Gressitt (1941) was brought to our attention. The publication date of this hitherto overlooked article by Gressitt was researched and the nomenclatural status of three taxa described in this article were studied. Two junior names are homonyms and three are junior objective synonyms, which we synonymize here.

We follow the International Code of Zoological Nomenclature Recommendation 21F (ICZN 1999) in publishing this study. We also provide a PDF of the Gressitt (1941) article as a Suppl. material 1 to allow for proper citation of the referenced taxa.

## ﻿Materials and methods

We follow the International Code of Zoological Nomenclature (ICZN 1999) to make decisions about proper use of the names published in Gressitt (1941) and to make appropriate corrections. The following articles are applicable to this paper.

Glossary: noun phrase, n.

primary homonym

Each of two or more identical specific or subspecific names established for different nominal taxa and originally combined with the same generic name [Art. 57.2]. For variant spellings deemed to be identical see Article 58.

objective synonym

Each of two or more synonyms that denote nominal taxa with the same name-bearing type, or (in the cases of family-group and genus-group taxa) that denote nominal taxa with name-bearing types whose own names are themselves objectively synonymous.

### ﻿Article 21. Determination of date

21.1. Date to be adopted

Except as provided in Article 3, the date to be adopted as the date of publication of a work and of a contained name or nomenclatural act is to be determined in accordance with the following provisions.

21.2. Date specified

The date of publication specified in a work is to be adopted as correct in the absence of evidence to the contrary.

21.3. Date incompletely specified

If the day of publication is not specified in a work, the earliest day on which the work is demonstrated to be in existence as a published work is to be adopted as the date of publication, but in the absence of such evidence the date to be adopted is

21.3.1. the last day of the month, when month and year, but not day, are specified or demonstrated, or

21.3.2. the last day of the year when only the year is specified or demonstrated.

21.4. Date incorrect

If the date of publication specified in a work is found to be incorrect, the earliest day on which the work is demonstrated to be in existence as a published work is to be adopted. In the absence of evidence as to day, the provisions of Article 21.3 apply.

**Recommendation 21F.** Correction of date. If an author of a new scientific name or other nomenclatural act is aware that the date specified in the work containing it is incorrect or incomplete, he or she should publish a correction in some suitable manner.

32.3. Preservation of correct original spelling

The correct original spelling of a name is to be preserved unaltered, except where it is mandatory to change the suffix or the gender ending under Article 34 (for treatment of emendations and incorrect subsequent spellings see Articles 32.5, 33.2, 33.3, 33.4).

32.4. Status of incorrect original spellings

An original spelling is an “incorrect original spelling” if it must be corrected as required in Article 32.5. An incorrect original spelling has no separate availability and cannot enter into homonymy or be used as a substitute name.

32.5. Spellings that must be corrected (incorrect original spellings)

32.5.1. If there is in the original publication itself, without recourse to any external source of information, clear evidence of an inadvertent error, such as a lapsus calami or a copyist’s or printer’s error, it must be corrected. Incorrect transliteration or latinization, or use of an inappropriate connecting vowel, are not to be considered inadvertent errors.

32.5.1.1. The correction of a spelling of a name in a publisher’s or author’s corrigendum issued simultaneously with the original work or as a circulated slip to be inserted in the work (or if in a journal, or work issued in parts, in one of the parts of the same volume) is to be accepted as clear evidence of an inadvertent error.

53.3. Homonyms in the species group

Two or more available species-group names having the same spelling are homonyms if they were originally established in combination with the same generic name (primary homonymy), or when they are subsequently published in combination with the same generic name (secondary homonymy) (for species-group names combined with homonymous generic names see Article 57.8.1).

57.2. Primary homonyms

Identical species-group names established for different nominal taxa when originally combined with the same generic name (see also Articles 11.9.3.2 and 57.8.1) are primary homonyms [Art. 53.3] and the junior name is permanently invalid (but see Article 23.9.5).

Scans of text and figures from Gressitt (1941) and the supplementary PDF (Suppl. material 1) were made by Yu-Tang Wang in 2022 from a printed copy of the “Sichuan University Museum. 2014. Journal of the West China Border Research Society, Unabridged photocopied Edition, 7: 3350–3363. Zhonghua Book Company, Beijjing, ISBN 978-7-101-10485-1” deposited in Library of Mianyang Normal University. Photographs of Gressitt (1942c) were taken from reprints deposited in Gerard Tavakilian’s office in the Muséum national d’Histoire naturelle (MNHN) with a Sony T30 camera in 2008. Insect specimens were photographed with a Canon EOS 7D with Canon 100 mm macro lens and layers were stacked using Helicon Focus v. 7 (Figs 8, 11), or were photographed using a Nikon DS-Ri2 camera mounted on a Nikon SMZ25 stereo microscope and layers were captured and stacked in the NIS-Elements software (Figs 7, 9). Materials studied are deposited in the following institutions, museums, or private collections: **IZCAS** = Institute of Zoology, Chinese Academy of Sciences, Beijing, China; **MYNU** = Invertebrate Collection of Mianyang Normal University, Mianyang, Sichuan, China; **NU** = University of Nanking (Nanjing University), Nanjing, China; **SYSU** = Sun Yat-Sen University, Guangzhou, Guangdong, China; **USNM** (= **NMNH**) = National Museum of Natural History (Smithsonian Institution), Washington DC, USA (formerly United States National Museum); **WCUU** = West China Union University, in history.

In addition, the following abbreviations were used: **ICZN** = International Code of Zoological Nomenclature; **TD** = type depository; **TL** = type locality.

## ﻿Results

### ﻿The publication date of Gressitt’s “Chinese longicorn beetles of the tribe Tetraopini (Coleoptera)”

The publication date of Gressitt’s “Chinese longicorn beetles of the tribe Tetraopini (Coleoptera)” is 1941. Although the front page indicates “Volume XII / Series B, Natural Sciences / 1940”, it was not published in 1940 as planned. Graham (1941) wrote “in April, 1941”, indicating that volume XII, section A was not printed before April, 1941. In his “Report of the Editor, Section A 1940–1941”, Kilborn (1941b) wrote “As soon as Volume XI is completed, the printing of Volume XII, Sections A and B will begin. Happily, Volume XIII…..”, which indicates that Volume XII was not printed before the report date, probably around May 1941. Graham (1942) wrote: “During the past year volumes XI, XII, and XIII have been published, and also Series B for the year 1940.” Thus, it is clear that Section B of Volume XII for the year 1940 was published in 1941, but we cannot know the exact publication date, which could have been any time from May to the end of 1941. According to Article 21.3.2 of the ICZN, the date to be adopted is 31 December 1941.

**Figures 1, 2.**
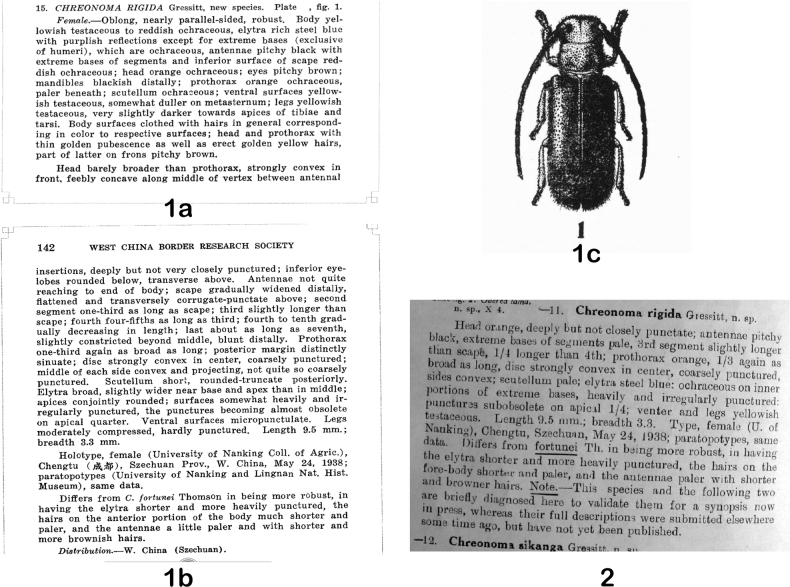
Descriptions of Bacchisa (Bacchisa) rigida (Gressitt, 1941) = *Chreonomarigida* Gressitt, 1941 **1a–c** senior homonym, original description by Gressitt (1941)**a** page 141 **b** page 142 **c** page 147 **2** junior homonym, redescription by Gressitt (1942c), page 6.

**Figures 3–6.**
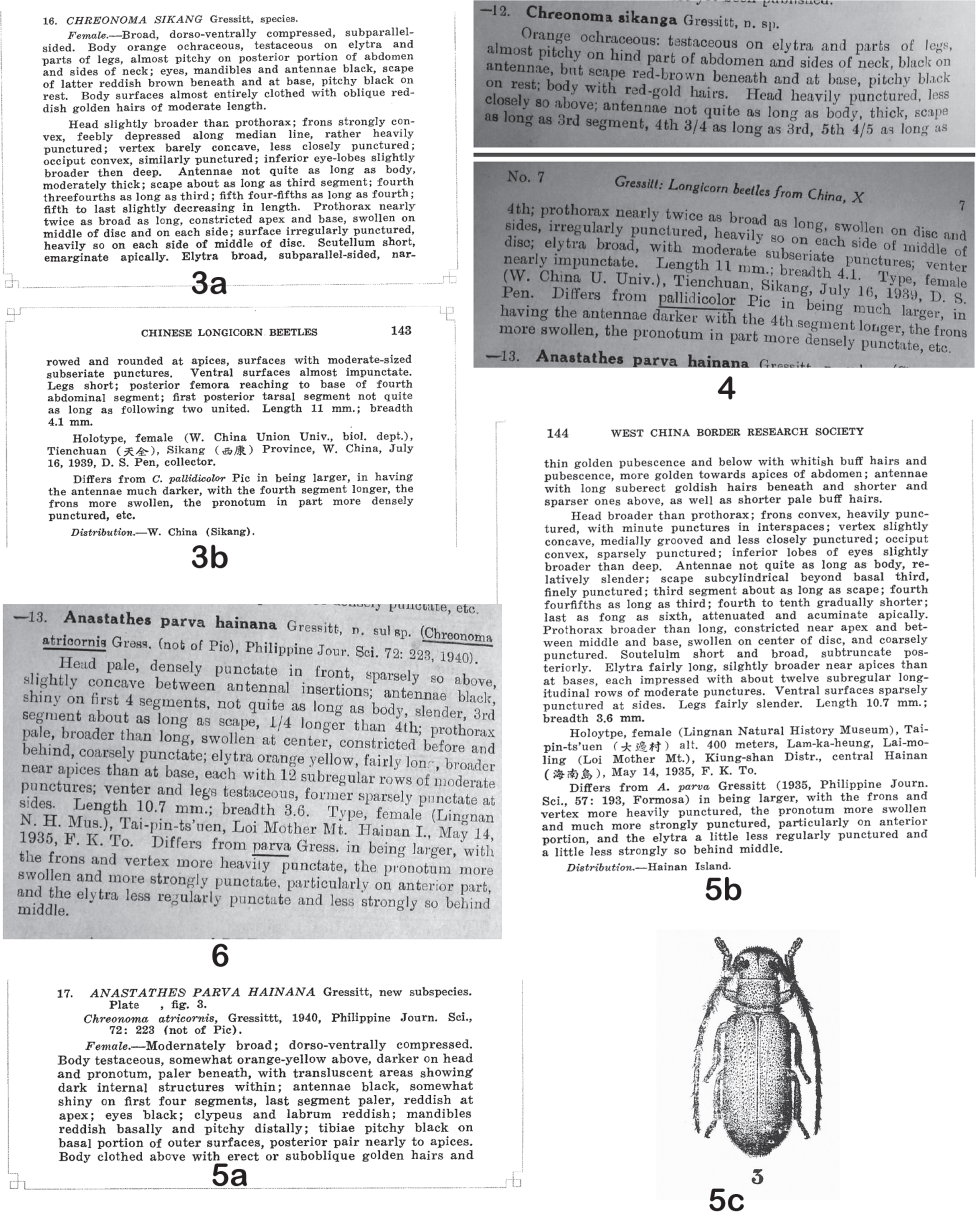
Descriptions **3, 4***Tetraophthalmussikang* (Gressitt, 1941) = *Chreonomasikang* Gressitt, 1941 **3a, b** senior homonym, original description by Gressitt (1941)**a** page 142 **b** page 143 **4** junior synonym, redescription by Gressitt (1942c), pages 6–7 **5, 6***Anastathesparvahainana* Gressitt, 1941 **5a–c** senior homonym, original description by Gressitt (1941)**a** page 143 **b** page 144 **c** page 147 **6** junior homonym, redescription by Gressitt (1942c), page 7.

The three new taxa published by Gressitt (1941) were published again in 1942 as if they were new (Gressitt 1942c), and this earlier paper had not been cited by Higa (1983); indeed, it has not been cited by anybody, including Gressitt himself (Gressitt 1951, 1957; Breuning 1956). This “missing” or “ignored” article by Gressitt (1941) was not indexed by the Zoological Record and Titan database (Tavakilian and Chevillotte 2022). Therefore, we show the original descriptions of the three taxa here in Figs 1, 3, and 5, and provide a PDF of Gressitt’s (1941) paper as Suppl. material 1.

### ﻿Photographic reproduction

The Unabridged, Photocopied Edition of the “Journal of the West China Border Research Society” was published in 2014 but has no taxonomic significance as it does not include any new taxa, new homonyms, or new synonyms (Sichuan University Museum 2014). It serves to highlight the original publication and possibly attract more current readers.

The same can be said of Gressitt’s “New longicorn beetles from China” series of publications. For example, “New longicorn beetles from China: VIII (Coleoptera: Cerambycidae)” (Gressitt 1942a) was originally published in “Lingnan Natural History Survey and Museum, Special Publication 2, 1–6” on 17 February 1942 (Evenhuis 2015), and photographically reproduced in “Lingnan Science Journal 21 (1–4), Supplement No. 2: 1–6” in 1945; “New longicorn beetles from China: IX (Coleoptera: Cerambycidae)” was originally published in “Lingnan Natural History Survey and Museum, Special Publication 3, 1–8” on 28 March 1942 (Evenhuis 2015), and photographically reproduced in “Lingnan Science Journal 21 (1–4), Supplement No. 3: 1–8” in 1945; and “New longicorn beetles from China: X (Coleoptera: Cerambycidae)” was originally published in “Lingnan Natural History Survey and Museum, Special Publication 7, 1–11” in 1942, and photographically reproduced in “Lingnan Science Journal 22 (1–4), Supplement No. 7: 1–11” on 31 March 1948. Nobody has since cited the photographic reproductions, and they were not listed in Higa’s (1983) bibliography of J.L. Gressitt.

### ﻿Taxonomy

#### ﻿ Bacchisa (Bacchisa) rigida

Taxon classificationAnimaliaColeopteraCerambycidae

(Gressitt, 1941)

EA301BB6-408D-5F1E-A1CC-F817DA503EC4

Figs 1 , 2 , 7 , 8

Chreonoma rigida Gressitt, 1941: 141, fig. 1. TL China: Sichuan. TDSYSU, ex NU.
Chreonoma rigida Gressitt, 1942c: 6. TL China: Sichuan. TDSYSU, ex NU. Homonym, syn. nov.
Chreonoma rigida : Gressitt 1942d: 42; Gressitt 1951: 616, 619.
Bacchisa (Bacchisa) rigida : Breuning 1956: 420, 436; Breuning 1966: 659; Löbl and Smetana 2010: 236; Lin and Yang 2019: 247; Danilevsky 2020: 331.
Bacchisa rigida : Hua 2002: 198; Hua et al. 2009: 203, 343, pl. LXIII, fig. 796.

##### Type material examined.

**Figures 7–11.**
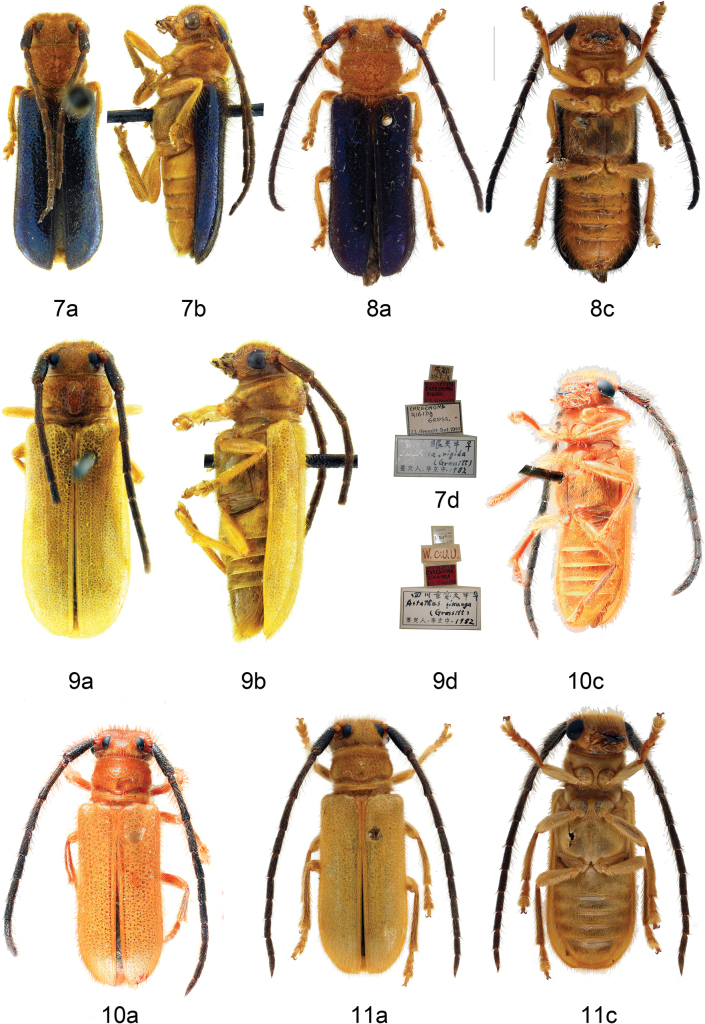
Habitus **7, 8***Bacchisarigida* (Gressitt, 1941) = *Chreonomarigida* Gressitt, 1941 **7** holotype, female, from Sichuan **8** female, from Sichuan **9***Tetraophthalmussikang* (Gressitt, 1941) = *Chreonomasikang* Gressitt, 1941, holotype, female, from Sichuan **10***Anastathesparvaparva* Gressitt, 1935, holotype, male, from Taiwan Island, photographed by Eugenio Nearns **11***Anastathesparvahainana* Gressitt, 1941, female, from Hainan Island **a** dorsal view **b** lateral view **c** ventral view **d** labels.

***Holotype***, female (Fig. 7), China Szechuan, Chengtu, 24 May 1938 (SYSU).

##### Other material examined.

**China Sichuan**: 1 female, Emeishan, Baoguosi, 28 July 1957, leg. Zong-Yuan Wang (IZCAS); 1 female (Fig. 8), Chengdu, 24 June 1981, leg. Yuan-Jiang Hui (IZCAS).

##### Distribution.

China: Sichuan.

##### Remarks.

*Chreonomarigida* Gressitt, 1942c is junior homonym and objective synonym of *Chreonomarigida* Gressitt, 1941. The Zhejiang record was firstly reported by Gressitt (1951) with a question mark; Breuning (1956, 1966) did not include it, while Hua (2002)Hua et al. (2009), Löbl and Smetana (2010), Lin and Yang (2019), and Danilevsky (2020) did include this Zhejiang record. We did not find Gressitt’s Zhejiang specimens in either IZCAS or SYSU. Although a photograph on the website http://bezbycids.com/byciddb/wdetails.asp?id=26554 is said to be a voucher specimen of *Bacchisarigida* examined by Gressitt (1951), this photograph is of a different species. Therefore, we remove Zhejiang from the list of localities from which *C.rigida* is known.

#### ﻿ Tetraophthalmus sikang

Taxon classificationAnimaliaColeopteraCerambycidae

(Gressitt, 1941)

FC882C61-07E5-51E1-A26A-1E2A402ED383

Figs 3 , 4 , 9

Chreonoma sikang Gressitt, 1941: 142. TL China: Sichuan (Sikang). TDSYSU ex WCUU.
Chreonoma sikanga Gressitt, 1942c: 6. TL China: Sichuan (Sikang). TDSYSU ex WCUU. Syn. nov.
Astathes sikanga : Gressitt 1942d: 42; Gressitt 1951: 620, 621; Hua 2002: 197; Hua et al. 2009: 201, 340, pl. LXVIII, fig. 775.
Astathes (Tetraophthalmus) sikanga : Breuning 1956: 511; Breuning 1966: 667.
Tetraophthalmus sikanga : Löbl and Smetana 2010: 237; Lin and Yang 2019: 249; Danilevsky 2020: 331.

##### Material examined.

***Holotype***, female (Fig. 9a–d), China, Szechuan, Tienchuan, Sikong, 16 July 1939, leg. D.S. Pen (SYSU).

##### Distribution.

China: Sichuan.

##### Remarks.

Gressitt (1941) spelled the new species epithet as “*sikang*”, which might be a typographical error. He clearly wrote “*CHREONOMA SIKANGA*” on the holotype label (Fig. 9d) and later (Gressitt 1942c) wrote “*sikanga*”, which indicates that he named this species “*sikanga*”, in the same way as *Anastathesparvahainana*. However, according to the Article 32.3 of the ICZN, the correct original spelling of a name is to be preserved unaltered. Additionally, Gressitt’s (1942c) spelling “*sikanga*” was not “issued simultaneously with the original work”, so that spelling cannot be considered as an “author’s corrigendum to be accepted as clear evidence of an inadvertent error” (ICZN Articles 32.4 and 32.5). Therefore, *Chreonomasikanga* Gressitt, 1942c is a junior and objective synonym of *Chreonomasikang* Gressitt, 1941.

###### ﻿*Anastathesparva* Gressitt, 1935

Figs 5, 6, 10, 11

#### ﻿ Anastathes parva parva

Taxon classificationAnimaliaColeopteraCerambycidae

Gressitt, 1935

B1E7CD48-6606-55DD-A4AE-901CEAD4F350

Fig. 10a, c

Anastathes parva Gressitt, 1935: 193. TL China: Taiwan. TDUSNM.
Anastathes parva parva : Gressitt 1951: 620; Hua 2002: 192; Chou 2004: 365, figs; Chou 2008: 365, figs.
Anastathes parva : Breuning 1956: 486; Breuning 1966: 664; Hua et al. 2009: 195, 334, pl. LXIII, fig. 725; Lingafelter et al. 2013: 136. fig. 72a; Lingafelter et al. 2014: 196, fig. 128g; Danilevsky 2020: 330 [in part].
Anastathes parvus [sic]: Löbl and Smetana 2010: 236 [in part]; Lin and Yang 2019: 245 [in part].

##### Type material examined.

***Holotype***, male (Fig. 10), China, central Formosa, Bukai, near Hori, alt. 1000 m, 1934.VI.12 (USNM 50901).

##### Distribution.

China: Taiwan.

#### ﻿ Anastathes parva hainana

Taxon classificationAnimaliaColeopteraCerambycidae

Gressitt, 1941

29DBB42C-F963-5792-A4AF-1ADA49DF965B

Figs 5 , 6 , 11

Anastathes parva hainana Gressitt, 1941:143, fig. 3. **TL** China: Hainan. **TD**SYSU.
Anastathes parva hainana Gressitt, 1942c: 7. **TL** China: Hainan. **TD**SYSU. Homonym, syn. nov.
Anastathes parva hainana : Gressitt 1942d: 43; Gressitt 1951: 620; Hua et al. 1993: 174, 308, pl. XXIV, fig. 403a, b; Hua 2002: 192; Hua et al. 2009: 195, 334, pl. LXIII, fig. 726.
Anastathes parva : Danilevsky 2020: 330 [in part].
Anastathes parva m. hainana: Breuning 1956: 487; Breuning 1966: 664.
Anastathes parvus [sic]: Löbl and Smetana 2010: 236 [in part]; Lin and Yang 2019: 245 [in part].

##### Other material examined.

**China Hunan**: 1 male, Yizhang, Mangshan, Tiantaishan, 15 July 2008, leg. Hong-Bin Liang (IZCAS). **Guangdong**: 1 male, Tso-kok-wan, Lungtau Shan, 250–350 m, 5 June 1947, L. Gressitt & T.S. Lam (SYSU, Ce-003756); 1 male, Laugtau Shan, Kuh-kiang District, 300 m, 7 July 1947, W.T. Tsang (SYSU, Ce-003764); 1 male, Ruyuan County, Nanling Nat. Rev., Xiaohuangshan, 1011 m, 24.9013°N, 113.0392°E, 18 July 2022, leg. Mei-Ying Lin & Chang-Peng Yan (MYNU); 1 female, Ruyuan County, Nanling Nat. Rev., Babaoshan, 1023 m, 24.9330°N, 113.0192°E, 17 July 2022, leg. Mei-Ying Lin & Chang-Peng Yan (MYNU). **Hainan**: 1 male 1 female, Diaoluoshan, 2–6 May 1965, leg. Si-Kong Liu (IZCAS); 1 male (Fig. 11), Diaoluoshan, 1000 m, 23 April 1980, leg. Shu-Yong Wang (IZCAS). **Guangxi**: 1 male, Longsheng, Neicujiang, 840 m, 6 June 1963, leg. Chun-Guang Wang (IZCAS); 1 male, Longsheng, Tianpingshan, 740 m, 3 June 1963, leg. Chun-Guang Wang (IZCAS); 2 males, Longsheng, Huaping, Hongtan to Cujiang, 7 August 2006, leg. Mei-Ying Lin (IZCAS); 1 male, Longsheng, Baiyan, 1150 m, 23 June 1963, leg. Shu-Yong Wang (IZCAS); 1 male, same data but 18 June 1963; 1 male, same data but 18 June 1963, leg. Yong-Shan Shi; 1 male, same data but leg. Chun-Guang Wang.

##### Distribution.

China: Zhejiang, Hunan, Fujian, Guangdong, Hainan, Guangxi. Vietnam.

##### Remarks.

*Anastathesparvahainana* Gressitt, 1942c is junior homonym and objective synonym of *Anastathesparvahainana* Gressitt, 1941. The holotype of *Anastathesparvahainana* Gressitt, 1941 is a female from Tai-pin-ts’uen, Loi Mother Mountain, Hainan Island, 1935.V.14, leg. F.K. To (SYSU). We did not find this holotype during our study, and it was not included in the iconography by Hua et al. (2009).

All authors have treated Gressitt (1942c) as the original description of *Anastathesparvahainana*, except Breuning (1956 and 1966), who wrongly cited Gressitt (1942d). Löbl and Smetana (2010) incorrectly treated the misidentification of the Hainan population by Gressitt (1940) “*Chreonomaatricornis*” as a homonym of *Chreonomaatricornis* Pic, 1922, and considered *hainana* Gressitt, 1942c as a replacement name of “*Chreonomaatricornis*” (Gressitt 1940), while Danilevsky (2020) erroneously considered *hainana* Gressitt, 1942c as a replacement name for *parva* Gressitt, 1935. Based on our study, *Anastathesparvahainana* Gressitt, 1942c is a duplicately published homonym of *Anastathesparvahainana* Gressitt, 1941, both based on the same holotype specimen, while Gressitt (1940) misidentified this species as *Chreonomaatricornis* Pic, 1922. We do not agree with Breuning (1956) who treated the Hainan Island population (Fig. 11) as an infrasubspecific variety of the Taiwan Island population (Fig. 10), but follow Gressitt (1941, 1942c, 1942d, 1951) in treating it as a subspecies.

In addition to the three taxa whose publication date is now known to have been one year earlier, Gressitt’s (1941) article moved *Astathesdioica* Fairmaire, 1878 to the genus *Chreonoma* one year earlier than Gressitt (1942d) and 10 years earlier than Gressitt (1951).

## ﻿Discussion

The “Journal of the West China Border Research Society” surely was an official publication. Some articles from this journal were indexed by the Zoological Record, although the Gressitt’s (1941) article was missing. The taxa published by Liu (1945) in this journal have been accepted as available and validly published, and in use by current herpetologists. For example, the genus *Vibrissaphora* Liu, 1945 and *V.boringii* Liu, 1945 have been cited by Rao et al. (2006).

On the distribution of the journal, Kilborn (1941a) wrote in his “Report of the Editor”: “Our exchange list continues to increase, showing that the Journal is being appreciated by many institutions in many lands.”

Gressitt did not receive the publication notification of the Tetraopini synopsis before he submitted his “New longicorn beetles from China: X” (Gressitt 1942c), which was published on 31 October 1942 (Evenhuis 2015). In this 1942 paper, following the description of “11. *Chreonomarigida* Gressitt, n. sp.”, he wrote: “Note.—This species and the following two are briefly diagnosed here to validate them for a synopsis now in press, whereas their full descriptions were submitted elsewhere some time ago, but have not yet been published” (Fig. 2). By the “following two” species, he meant “12. *Chreonomasikanga* Gressitt, n. sp.” (Fig. 4) and “13. *Anastathesparvahainana* Gressitt, n. subsp.” (Fig. 6). We do not know the exact date when Gressitt submitted “New longicorn beetles from China: X”, but it probably was after the publication date of “New longicorn beetles from China: IX” (Gressitt 1942b), which was 28 March 1942.

Gressitt (1942d, which was published in November 1942) wrote “dioica (Fairm.) new comb.” on page 43, indicating he still did not know of the publication of Gressitt (1941). Gressitt (1951) did not cite his 1941 paper but instead cited his 1942 paper. Even 10 years later, he probably still did not know that the 1941 paper had been published (Gressitt 1951).

We suspect that the other taxonomic paper in the same volume of the Journal of the West China Border Research Society, on Cercopidae (Liu 1941), has also been ignored by taxonomists. Three new species were published in it.

## Supplementary Material

XML Treatment for Bacchisa (Bacchisa) rigida

XML Treatment for Tetraophthalmus sikang

XML Treatment for Anastathes parva parva

XML Treatment for Anastathes parva hainana

## ﻿Additional information

### Author ORCIDs

Mei-Ying Lin https://orcid.org/0000-0001-9219-724X

Steven W. Lingafelter https://orcid.org/0000-0001-6294-4259

### Data availability

All of the data that support the findings of this study are available in the main text or Supplementary Information.

## Supplementary materials

Supplementary material 1 Chinese longicorn beetles of the tribe Tetraopini (Coleoptera) Data type original pdf Explanation note Gressitt, J. L. 1941. Chinese longicorn beetles of the tribe Tetraopini (Coleoptera). Journal of the West China Border Research Society, 12B [1940]: 134–147. This article were overlooked by all researchers, so we share the pdf. https://binary.pensoft.net/file/870752This dataset is made available under the Open Database License (http://opendatacommons.org/licenses/odbl/1.0/). The Open Database License (ODbL) is a license agreement intended to allow users to freely share, modify, and use this Dataset while maintaining this same freedom for others, provided that the original source and author(s) are credited.J. Linsley Gressitt
