# Supplementary material for: ﻿A hitherto overlooked article by Gressitt in 1941 (Insecta, Coleoptera, Cerambycidae, Lamiinae)
Source: Zookeys. 2023 Jun 29;1168:179–92. doi: 10.3897/zookeys.1168.107021 (PMC10838559; doi:10.3897/zookeys.1168.107021)
Supplement: Supplementary material 1 — Chinese longicorn beetles of the tribe Tetraopini (Coleoptera) [file zookeys-1168-179_article-107021__-s001.pdf]

JOURNAL

of the

WEST CHINA BORDER RESEARCH SOCIETY

華西邊疆研究學會雜誌

Volume XII

Series B, Natural Sciences

1940

Vol.XII B
第十二卷B 1940
3211

JOURNAL

of the

WEST CHINA BORDER RESEARCH SOCIETY

Series B
Natural Sciences

Editor: K. J. FISHER
(Mrs. H. L. Richardson).

Volume XII
1940

Vol.XII B
第十二巻B 1940
3213

CONTENTS

	PAGE
Editor's Foreword	5
Tadpoles of West China Salientia ....CH'ENG-CHAO LIU	7
Notes on the Life History and Breeding Habits of Gekko Japonicus and Gekko Subpalmitus ..C. C. LIU AND S. C. HU	63
On the Chinese Members of the Genus Clovia (Cercopidae, Homoptera) ....GAINES KAN-CHIH LIU	71
China-Root—Fu-Ling 茯苓 & T'u- Fu–Ling 土茯苓 A Problem in Chinese Medicinal Plants. ....S. Y. HU	80
A Preliminary List of Fresh Water Protozoa of Kiating, Szechwan ....H. ZANYIN GAW	87
Cytological Studies of a Haploid Wheat Plant C. H. LI AND H. W. LI	94
Soil and Man in West China ....H. L. RICHARDSON	102
Chinese Longicorn Beetles of the Tribe Tetraopini (Coleoptera) ....J. LINSLEY GRESSITT	134
A Butterfly Migration in Western Szechwan ....K. J. FISHER	148
NOTES AND OBSERVATIONS	152
Flight of Sea-gulls ....R. A. WALKER	152
Butterfly Migration ....W. SMALL	153

Vol.XII B
第十二卷B 1940
3215

EDITOR'S FOREWORD.

This volume marks a new departure in the publication of the Society's Journal. Following the example of more distinguished bodies, we are bringing out the Journal in two parts, Series A, General, and Series B, Natural Sciences. It is felt that this will not only be a convenience to members of the Society whose bent lies more particularly in one direction or the other, but that it will also facilitate the exchange of journals with other scientific societies. It is a welcome sign of the interest taken in our work that sufficient papers of a biological nature have been received to permit of the publication of the present number, and it is hoped that in future it will be possible to continue the practice.

Most of the papers in the present volume are of a technical nature. We trust that there will continue to be a supply of such works, and that the journal may be considered a worthy organ in which the results of professional scientific work may be published, and that our journal may form a valuable repository for information of an authoritative nature about the fauna and flora of the border countries. Nevertheless, it would be a great pity if the amateur naturalist were to be deterred from submitting the results of his own research and observations, since these, if accurately made and carefully recorded, are often important in extending, and sometimes correcting, the work done in laboratories. It should never be forgotten that the entire science of Biology was begun by amateurs; indeed, had it not been for the devoted work of the old "naturalists" of the seventeeth and eighteenth centuries, it is improbable that it would ever have reached the development that we see to-day. And the modern professional scientific worker is not only still indebted to the workers of the past, but also to present-day amateur naturalists, who collect in their spare time information which is of the utmost value to all.

Phenological studies, that is the recording each year of the first appearance of common birds or insects, or of the flowering of wild plants, may be of the greatest value when continued over a number of years. The working out of the connection between weather records and the arrival or departure of migrant birds, etc., is not only of great intellectual interest, but may be of value to the agriculturalist. In the section headed "NOTES AND OBSERVATIONS", this journal will always be glad to report such facts as: the date on which the various local cuckoos are first heard (the "brain fever" bird, etc.), the appearance of the hoopoe or other occasional visitors, notes on years of unusual prevalence or scarcity of

Vol.XII B
第十二巻B 1940
3217

insect species, migrations of butterflies or moths seen in West China, or such other notes as may interest readers and contributors.

The present writer once had occasion to look through European biological journals of the past hundred and fifty years for records of the appearance of a moth which, very rare in the British Isles, sometimes occurs as a minor pest in southern Europe. (*Celerio livornica*). From the sum of many years of such observations, almost wholly those of amateurs, it was possible to draw some extremely interesting conclusions as to the nature, and even, tentatively, the causes, of the intermittent appearance of this moth, and to find parallels with the appearance of a very closely related species in the United States. It is certain that the early naturalist, when he wrote to the editor of his favoured Journal to tell him of his "interesting experience in catching a slightly damaged specimen of that attractive rarity, *C. livornica,* as far north as Gloucestershire" did not imagine that he would, long after his death, be helping to throw light on such a subject as insect migration. Perhaps the Biologists of a century from now, while being slightly amused by the theories popular in our time, may still be very glad to find the *facts,* carefully recorded, by the amateur contributors to our columns.

K.J.F.R.

CHINESE LONGICORN BEETLES OF THE TRIBE TETRAOPINI (COLEOPTERA)*

J. LINSLEY GRESSITT

Lingnan Natural History Survey and Museum, Lingnan University.

This tribe of beetles, which stands almost at the end of the subfamily Lamiinae, is distributed in all the continents of the world except Australia and South America, but is particularly dominant in the Oriental Region, where eleven of the sixteen genera occur. In China, with the northward extention of this tropical region, five of the Oriental genera, besides the single Palearctic genus, are to be found. With the three additional new species described in this paper, the total number of species known from China now becomes twenty-one, or nearly one-tenth of the world's species. Most of these are fund in the South, seven being known from Hainan Island and also seven from mainland Kwangtung and Hong Kong. Very few extend north of the Yangtze in eastern China, but four are now known from Szechuan, an additional one from Sikang, and still another from Mongolia.

The new species herein described are from the collections of the University of Nanking, West China Union University, and the Lingnan Natural History Museum, and the type specimens are to be deposited in the respective collections. I am indbted to Prof. Shen Tseng, Miss K. O. Victoria Lieu, Prof. B. A. Slocum, Mr. C. S. Tsi, Prof. Chang Min-chuen, Miss D. S. Pen Dr. Sicien Chen and Dr. Gaines Liu in connection with this study.

Tribe TETRAOPINI

This tribe is distinguished from others of the Lamiinae by the fact that its species have the eyes each divided into two distant lobes connected by a fine thread, and by the tarsal claws being divaricate and toothed basally. The species are mostly broad and oblong, flattened above and sometimes widened posteriorly. They are generally yellow and black or metallic bluish.

KEY TO THE CHINESE GENERA OF TETRAOPINI

1. Metasternum with a process projecting anteriorly between middle coxae 2
   Metasternum lacking a process projecting anteriorly between middle coxae 3

---

*Contribution from the Lingnan Natural History Survey and Museum, Lingnan University.

2. Intercoxal process of metasternum extending forward and overlapping apex of mesosternal process, which is subvertical .... *Astathes*
Intercoval proces of metasternum meeting mesosternal process at about middle of coxae, and entering its angular emargination .... *Anastathes*

3. Metepisternum fairly broad 4
Metepisternum very narrow; body small and slender, more or less cylindrical .... *Tetrops*

4. Antennae generally shorter than body in female; scape not reaching to middle of prothorax; sides of head without projecting fringes of hair 5
Antennae longer than body in female; scape reaching beyond middle of prothorax; sides of head with projecting fringes of hair, particularly in male .... *Lasiophrys*

5. Elytra broadly expanded behind middle; antennae slender, with second segment about one-quarter as long as scape .... *Plaxomicrus*
Elytra nearly parallel-sided; antennae moderately stout, with second segment generally one-third as long as scape, or longer .... *Chreonoma*

Genus *TETROPS* Stephens

Tetrops Stephens, 1831, Ill. Brit. Ent. Mand., 4: 228, 241; Lacordaire, 1872, Gen. Col., 9: 872, 880.

Metasternum very narrow; form slender. Antennae slender, shorter than body in both sexes; head convex in front, hardly concave between antennal insertions; prothorax cylindrical, distinctly grooved basally; elytra parallel-sided, subtruncate posteriorly. The species are very small and are mostly black and pale yellow in color.

Genotype.—*Leptura praeusta* Linnaeus.

Range.—Holarctica.

1. *TETROPS HAUSERI* Reitter.

Tetrops hauseri Reitter, 1897, Deutsche Ent. Zeits.: 225 (Tianshan).

I have seen no material of this species, which is not found in China proper.

Distribution.—Mongolia (Tian Shan)

Genus *LASIOPHRUS* Gahan

Lasiophrus Gahan, 1901, Trans. Ent. Soc. London: 71; Gressitt, 1940, Philippine Journ. Sci., 72: 226.

Head with lateral fringes of hairs, particularly in male antennae longer than body in both sexes; scape reaching beyond middle of prothorax; prothorax transverse, constricted near base; elytra parallel-sided, rounded apically: metasternum with a process extending anteriorly a short distance between middle coxae.

Vol.XII B 第十二巻B 1940 3351

Genotype.—*Lasiophrys latifrons* Gahan.

Range.—Indo-Chinese Subregion.

2. *LASIOPHERYS TINHOSENSIS* Gressitt.

Lasiophrys tinhosensis Gressitt, 1940, Philippine Journ. Sci., 72: 226, pl. 6, fig. 13 (Tinhosa, near Hainan I.).

Orange ochraceous; antennae black; apical quarter of elytra brownish black; body surfaces with erect orange hairs and thin golden pubescence. Frons deeply but sparsely punctured; pronotum densely punctured on each side of disc; elytra with deep punctures, mostly in regular longitudinal rows. Length 10.2-10.8 mm.; breadth 3.4-4.

Distribution.—Tinhosa (Taai-chau I.), off Hainan Island.

Genus *PLAXOMICRUS* Thomson

Plaxomicrus Thomson, 1857, Archives Ent., 1: 57; Lacordaire, 1872, Gen. Col., 9: 672, Gahan, 1901, Trans. Ent. Soc. London: 69.

Head broader than prothorax; antennae relatively slender, shorter than body in female; prothorax strongly swollen at each side; elytra broadly expanded behind middle, rounded posteriorly.

Genotype.—*Plaxomicrus ellipticus* Thomson.

Range—Indo-Chinese and Indo-Malayan Subregions.

KEY TO THE CHINESE SPECIES OF PLAXOMICRUS

1. Elytra not entirely metallic blue, violaceous or purplish, at least partly testaceous apically; elytra not very broadly expanded behind middle .... *guerryi*

Elytra entirely metallic violet blue with purplish reflections, broadly expanded behind middle .... *ellipticus*

3. *PLAXOMICRUS ELLIPTICUS* Thomson. Plate , fig. 4.

Plaxomicrus ellipticus Thomson, 1857, Archives Ent., 1: 58 (Shanghai).

Male.—Body largely testaceous yellow to pale orange ochraceous and elytra metallic purplish blue: head pale ochraceous; antennae with first two segments ochraceous, distal portions pitchy above, third testaceous with extreme apex blackish, fourth testaceous proximally and blackish distally, fifth and following segments black; prothorax and scutellum pale ochraceous; ventral surfaces testaceous, largely reddish or pitchy on sides of metasternum and abdomen; legs testaceous. Head and prothorax irregularly punctured, the latter more densely so; antennae barely longer than body; elytra very sparsely punctured, beset with suberect black hairs, densest on external margins. Length 10.8 mm.; breadth 5.2

Some specimens (University of Nanking Coll. of Agric.; and Gressitt coll.) were collected at Chengtu (成都), Szechuan,

華西邊疆研究學會雜誌
3352

W. China in early July, 1938, by K. F. Chen. New to Szechuan Province.

Distribution.—Kiangsu (Shanghai); Szechuan (Chengtu).

4. *PLAXOMICRUS GUERRYI* (Pic).

Asthates Guerryi Pic, 1911, Matériaux Longicornes, 8 (1): 20 (Yunnan).

Asthates aplicalis Pic, 1911, l.c. (Yunnan), new synonym.

Plaxomicrus Guerryi Pic, 1912, Materiaux Longicornes, 8 (2): 22; Aurivillius, 1923, Coleopt. Catal. 74: 578.

Plaxomicrus apicalis, Pic, 1912, Materiaux Longicornes, 8 (2): 22.

Plaxomicrus Guerryi ab. *apicalis,* Plavilstshikov, 1916, Rev. Russe d'Ent., 15: 80; Aurivillius, 1923, Coleopt. Catal., 74: 578.

Body testaceous, metallic violaceous blue on basal halves, or less, of elytra, partly, or largely, pitchy, or purplish black, on ventral surfaces; long pale or pitchy hairs on most of body surfaces; antennae testaceous with distal halves more or less brownish, or completely pitchy black, as long as body in male and slightly shorter in female. Elytra subparallel-sided, feebly punctured, with a few larger punctures basally. Length 12-14 mm.

Since Pic's *apicalis* was obviously founded merely on a slight color variation, and probably came from the same general locality as *guerryi,* I feel that the former has no valid systematic status, and must be considered a synonym of *guerryi.*

I have studied a specimen in the British Museum collected at Yei-chin, alt. 6,400 ft., Mekong River, Yunnan, June 20, 1922, by Prof. J. W. Gregory. This specimen has only the basal quarters of the elytra metallic, the antennae entirely pitchy black and the metathorax and first four abdominal seaments purplish black.

Distribution.—Yunnan.

Genus *CHREONOMA* Pascoe

Chreonoma Pascoe, 1867, Trans. Ent. Soc. London, (3) 3: 348, 358; Lacordaire, 1872, Gen. Col. 9: 872, 876; Gahan, 1901, Trans. Ent. Soc. London: 63; Gressitt, 1940, Philippine Journ. Sci., 72: 223.

Body form moderately broad, suboblong; head as broad as, or brader than, prothorax, convex in front; inferior lobes of eyes a little broader than deep; antennae shorter, or very little longer, than body; prothorax transverse, swollen above and at sides; elytra broadly rounded apically; mesosternal intercoxal process narrow, gradually declivitous anteriorly.

Genotype.—*Chreonoma venusta* Pascoe.

Range.—Oriental and southeastern Palearctic Regions.

Vol.XII B
第十二巻B 1940
3353

KEY TO THE CHINESE SPECIES OF CHREONOMA

1. Elytra entirely blue or violaceous 2
   Elytra at least partly testaceous 5

2. Legs entirely testaceous 3
   Tarsi and apices of tibiae black .... *atritarsis*

3. Antennae largely black 4
   Antennae testaceous on at least first several segments; hairs on fore-body pale and short; elytra heavily punctured .... *hoffmanni*

4. Hairs on fore-body dark and very long; elytra heavily punctured .... *fortunei*
   Hairs on fore-body mostly pale and not very long; elytra heavity and not very closely punctured .... *rigida*

5. Elytra entirely testaceous 6
   Elytra not entirely testaceous 7

6. Fourth antennal segment three-fiifths as long as third; antennae ochraceous .... *pallidicolor*
   Fourth antennal segment four-fifths as long as third; antennae blackish .... *sikanga*

7. Only extreme bases of elytra pale 8
   Elytra with either basal halves or apical halves, or equivalent, pale 9

8. Elytra testaceous basally for equivalent of one-half length of prothorax; head of male lacking frontal carina; abdomen densely hairy below .... *comata*
   Elytra testaceous basally for equivalent of one-fourth length of prothorax; head of male with a frontal carina; abdomen sparsely hairy below .... *basalis*

9. Elytra with regular longitudinal rows of large punctures ... 10
   Elytra with sparse irregular punctures, or small, close subregular ones 11

10. Only apical third of elytra metallic; remainder orange testaceous .... *cyaneoapicalis*
    Nearly apical half of elytra metallic; remainder reddish ochraceous .... *cyaneoapicalis dimidiata*

11. Basal portions of elytra testaceous; elytral punctures small and close; body slender; less than 10 millimeters in length .... *dicica*
    Basal portions of elytra bluish black; elytral punctures moderately large and sparse; body broad, more than 12 millimeters in length .... *nigronotata*

5. *CHREONOMA ATRITARSIS* Pic.

Chreonoma atritarsis Pic, 1912, Materiaux Longicornes, 8 (2): 21, (China?); Gressitt, 1935, Philippine Journ. Sci., 57: 192 (Formosa); 1937, Lingnan Sci. Journ., 16: 620 (Kiangsi and Kwangtung); 1939, 18: 111.

Orange testaceous; elytra metallic violet blue to greenish blue; antennae black with base of scape ochraceous and most of third and fourth, and bases of fifth to seventh, segments testaceous; tarsi and apices of tibiae black. Elytra deeply and irregularly punctured on basal twothirds. Length 9-12.5 mm.

Several specimens were collected at Meitan (湄潭), N. Kweichow (貴州) Province, spring of 1940, by Dr. Liu Kan-chih. New to Kweichow.

Distribution.—Kiangsi; Kweichow; Kwangtung; Formosa.

6. *CHREONOMA BASALIS* Gahan.

Chreonoma basalis Gahan, 1894, Trans. Ent. Soc. London: 487 (Hongkong); Gressitt, 1939, Lingnan Sci. Journ., 18: 111, 112; 1940, Philippine Journ. Sci., 72: 224.

Ochraceous; elytra violet or deep blue with a narrow ochraceous band at extreme base; last few antennal segments blackish; tarsi and tibiae completely ochraceous. Elytra with deep and moderately sparse punctures on basal two-thirds, partly arranged in longitudinal lines. Length 10-13 mm.

Distribution.—Hong Kong; Kwangtung; Hainan.

7. *CHREONOMA COMATA* Gahan.

Chreonoma comata Gahan, 1901, Trans. Ent. Soc. London: 67, pl. 4. fig. 8; Gressitt, 1939, Lingnan Sci. Journ., 18: 112.

Body testaceous, more yellowish on head, pronotum and bases of antennae; elytra violet blue except for a basal yellowish band of about the width of half length of prothorax; antennae blackish brown on last six segments and appices of fourth and fifth; ventral surfaces of body, and legs, with tawny hairs, which are longest on sides of abdomen. Elytra closely punctured, the punctures smaller posteriorly; third to eighth or ninth antennal segments slightly angulate actoapically. Length 12 mm.

Distribution.—Hong Kong.

8. *CHREONOMA CYANEOAPICALIS* Gressitt.

Chreonoma cyaneoapicalis Gressitt, 1939, Lingnan Sci. Journ., 18: 112, pl. 2, fig. 11 (Fong-tong-ping, N. Kwangtung).

Yellowish testaceous; apical two-sevenths of elytra steel blue with a greenish tinge; antennae pitchy black on apical halves; head reddish in front, Body broad; antennae fairly stout; pronotum strongly swollen and heavily punctured in center; elytra with moderately small punctures arranged in fairly regular longitudinal rows. Length 6-8.5 mm.

Distribution —N. Kwangtung.

9. *CHREONOMA CYANEOAPICALIS DIMIDIATA* Gressitt.

Chreonoma cyaneoapicalis dimidiata Gressitt, 1940, Philippine Journ. Sci., 72: 224 (Hainan).

Body ochraceous; elytra steely violet or purplish blue on apical four-ninths; antennae pitchy brown on distal segments and slightly pitchy on middle segments; legs testaceous. Pronotum moderately swollen and moderately punctured on center;

Vol.XII B 第十二卷B 1940 3355

elytra with longitudinal rows of narrow punctures. Body more slender than in the typical form. Length 6.6 mm.

Distribution.—Hainan Island.

10. *CHREONOMA DIOICA* (Fairmaire), new combination. Plate , fig. 2.

Astathes dioica Fairmaire, 1878, Ann. Soc. Ent. France, (5) 8: 133 (Central China).

Pale testaceous, slightly ochraceous on head and pronotum; elytra with the discs purplish, the apical halves steel blue, or completely testaceous; prothorax with a slight pitchy spot on each side of disc and another below middle of each side; antennae largely pitchy black, except on undersides of basal segments; metathorax and first four abdominal segments shiny pitchy black; pro-and mesosterna and sides of femora pichy. Front of head closely punctulate; pronotum strongly swollen along middle, heavily punctured; elytra finely and closely punctured in an irregular manner. Length 8.4-9 mm.

Several specimens were collected or reared from apple (*Malus*) at Cheng-tu (成都), Szechuan Prov., W. China in April and May, 1937, by Prof. Shen Tseng, and in the spring of 1940 by Prof. Tseng and Miss K. O. Victoria Lieu.

Host plant.—Malus Communis (Chengtu).

Distribution.—Szechuan; central China.

11. *CHREONOMA PORTUNEI* (Thomson).

Plaxomicrus fortunei Thomson, 1857, Archives Ent., 1: 58, pl. 8, fig. 2.

Chreonoma fortunei Gahan, 1901, Trans. Ent. Soc. London: 68; Ganglbauer, 1887, Horae Soc. Ent. Ross., 20: 132; Okamoto, 1927, Ins. Matsumurana, 2: 82; Savio, Notes d'Ent. Chinoise, 1 (4): 5-7, figs. 1-3 (biology); Matsushita, 1933, Journ. Fac. Agr. Hokkaido Imp. Univ., 34: 429.

Body yellowish testaceous; elytra entirely steel blue; proximal portions of first four antennal segments testaceous, sometimes darker; each side of metasternum with a black spot. Anterior portion of body with long erect black or yellowish hairs. Elytra heavily and somewhat closely punctured. Length 9-10 mm.

Host plants.—Photinia serrulata Lindley (Shanghai); *Prunus* (Japan).

Distribution.—Kiangsu; Anhwei; Chekiang; Korea; Japan.

12. *CHREONOMA HOFFMANNI* Gressitt.

Chreonoma hoffmanni Gressitt, 1939, Lingnan Sci. Journ., 18: 113, pl. 2, fig. 12 (Taam-yuen-tung, N. Kwangtung).

Body ochraceous; elytra steel blue; antennae slightly pitchy brown, with brownish hairs, on last four or five segments and apex of scape; legs ochraceous; front of head

reddish ochraceous. Anterior portion of body with meager erect golden buff hairs and golden pubescence. Pronotum swollen, with punctures on each side of swelling; elytra moderately punctured in subregular longitudinal rows. Length 11-11.5 mm.

Distribution. N. Kwangtung.

13. *CHREONOMA NIGRONOTATA* Pic.

Chreonoma nigronotata Pic. 1912, Materiaux Longicornes, 8: 21 ("Tsouglov", Asia); Gressitt, 1937, Lingnan Sci. Journ., 16: 621 (Kiangsi); 1939. 18: 114 (Kwangtung).

Body orange testaceous, paler on apical halves of elytra; basal halves of elytra black with violet or purplish tinges; antennae with apices of second to sixth segments, and most of last five segments, pitchy black. Pronotum with long erect ochraceous hairs, strongly swollen and somewhat finely rugulose-punctate; elytra impressed with relatively small scattered punctures. Length 14-15 mm.

Distribution.—Kiangsi; Kwangtung; "Tsouglov".

14. *CHREONOMA PALLIDICOLOR* Pic.

Chreonoma pallidicolor Pic, 1916, Mel. Exot. Ent., 17: 6, Saigon; Gressitt, 1940, Philippine Journ. Sci., 72: 225 (Hainan).

Body pale yellowish testaceous; head, pronotum and basal portions of antennae ochraceous; distal antennal segments pitchy brown. Dorsal surfaces of body with erect golden buff hairs. Pronotum feebly swollen, with moderately heavy sparse punctures; elytra with fairly large punctures in subregular longitudinal rows on basal three-fifths. Length 8.3-11 mm.

Distribution.—Cochin-China; Hainan Island.

15. *CHREONOMA RIGIDA* Gressitt, new species. Plate , fig. 1.

Female.—Oblong, nearly parallel-sided, robust. Body yellowish testaceous to reddish ochraceous, elytra rich steel blue with purplish reflections except for extreme bases (exclusive of humeri), which are ochraceous, antennae pitchy black with extreme bases of segments and inferior surface of scape reddish ochraceous; head orange ochraceous; eyes pitchy brown; mandibles blackish distally; prothorax orange ochraceous, paler beneath; scutellum ochraceous; ventral surfaces yellowish testaceous, somewhat duller on metasternum; legs yellowish testaceous, very slightly darker towards apices of tibiae and tarsi. Body surfaces clothed with hairs in general corresponding in color to respective surfaces; head and prothorax with thin golden pubescence as well as erect golden yellow hairs, part of latter on frons pitchy brown.

Head barely broader than prothorax, strongly convex in front, feebly concave along middle of vertex between antennal

Vol.XII B
第十二卷B 1940
3357

insertions, deeply but not very closely punctured; inferior eye-lobes rounded below, transverse above. Antennae not quite reaching to end of body; scape gradually widened distally, flattened and transversely corrugate-punctate above; second segment one-third as long as scape; third slightly longer than scape; fourth four-fifths as long as third; fourth to tenth gradually decreasing in length; last about as long as seventh, slightly constricted beyond middle, blunt distally. Prothorax one-third again as broad as long; posterior margin distinctly sinuate; disc strongly convex in center, coarsely punctured; middle of each side convex and projecting, not quite so coarsely punctured. Scutellum short, rounded-truncate posteriorly. Elytra broad, slightly wider near base and apex than in middle; apices conjointly rounded; surfaces somewhat heavily and irregularly punctured, the punctures becoming almost obsolete on apical quarter. Ventral surfaces micropunctulate. Legs moderately compressed, hardly punctured. Length 9.5 mm.; breadth 3.3 mm.

Holotype, female (University of Nanking Coll. of Agric.), Chengtu (成都), Szechuan Prov., W. China, May 24, 1938; paratopotypes (University of Nanking and Lingnan Nat. Hist. Museum), same data.

Differs from *C. fortunei* Thomson in being more robust, in having the elytra shorter and more heavily punctured, the hairs on the anterior portion of the body much shorter and paler, and the antennae a little paler and with shorter and more brownish hairs.

Distribution.—W. China (Szechuan).

16. *CHREONOMA SIKANG* Gressitt, species.

Female.—Broad, dorso-ventrally compressed, subparallel-sided. Body orange ochraceous, testaceous on elytra and parts of legs, almost pitchy on posterior portion of abdomen and sides of neck; eyes, mandibles and antennae black, scape of latter reddish brown beneath and at base, pitchy black on rest. Body surfaces almost entirely clothed with oblique reddish golden hairs of moderate length.

Head slightly broader than prothorax; frons strongly convex, feebly depressed along median line, rather heavily punctured; vertex barely concave, less closely punctured; occiput convex, similarly punctured; inferior eye-lobes slightly broader then deep. Antennae not quite as long as body, moderately thick; scape about as long as third segment; fourth threefourths as long as third; fifth four-fifths as long as fourth; fifth to last slightly decreasing in length. Prothorax nearly twice as broad as long, constricted apex and base, swollen on middle of disc and on each side; surface irregularly punctured, heavily so on each side of middle of disc. Scutellum short, emarginate apically. Elytra broad, subparallel-sided, nar-

華西邊疆研究學會雜誌
3358

rowed and rounded at apices, surfaces with moderate-sized subseriate punctures. Ventral surfaces almost impunctate. Legs short; posterior femora reaching to base of fourth abdominal segment; first posterior tarsal segment not quite as long as following two united. Length 11 mm.; breadth 4.1 mm.

Holotype, female (W. China Union Univ., biol. dept.), Tienchuan (天全), Sikang (西康) Province, W. China, July 16, 1939, D. S. Pen, collector.

Differs from *C. pallidicolor* Pic in being larger, in having the antennae much darker, with the fourth segment longer, the frons more swollen, the pronotum in part more densely punctured, etc.

Distribution.—W. China (Sikang).

Genus *ANASTATHES* Gahan

Anastathes Gahan, 1901, Trans. Ent. Soc. London: 60; Gressitt, 1940, Philippine Journ. Sci., 72: 221.

Form stout, subrectangular; head broader than prothorax; antennae thick and short, third segment no longer than scape; prothorax strongly swollen above and at sides; elytra broad, parallel-sided; metasternum produced anteriorly to about middle coxae, entering angulate posterior emargination of mesosternal intercoxal process, which is almost vertical.

Genotype.—*Astathes nigricornis* Thomson.

Range.—Eastern part of Oriental Region.

KEY TO THE CHINESE SPECIES OF ANASTATHES

Body rather long and narrow; antennae relatively slender; pronotum with a moderate swelling at center and clothed with pubescence; elytra with a pair of feebly raised lines ....*parva hainana*

Body very broad; antennae stout; pronotum with a large swollen area reaching nearly to apex and base, almost lacking pubescence; elytra with two broad, distinctly raised longitudinal lines .... *robusta*

17. *ANASTATHES PARVA HAINANA* Gressitt, new subspecies. Plate , fig. 3.

Chreonoma atricornis, Gressittt, 1940, Philippine Journ. Sci., 72: 223 (not of Pic).

Female.—Modernately broad; dorso-ventrally compressed. Body testaceous, somewhat orange-yellow above, darker on head and pronotum, paler beneath, with transluscent areas showing dark internal structures within; antennae black, somewhat shiny on first four segments, last segment paler, reddish at apex; eyes black; clypeus and labrum reddish; mandibles reddish basally and pitchy distally; tibiae pitchy black on basal portion of outer surfaces, posterior pair nearly to apices. Body clothed above with erect or suboblique golden hairs and

Vol.XII B
第十二卷B 1940
3359

thin golden pubescence and below with whitish buff hairs and pubescence, more golden towards apices of abdomen; antennae with long suberect goldish hairs beneath and shorter and sparser ones above, as well as shorter pale buff hairs.

Head broader than prothorax; frons convex, heavily punctured, with minute punctures in interspaces; vertex slightly concave, medially grooved and less closely punctured; occiput convex, sparsely punctured; inferior lobes of eyes slightly broader than deep. Antennae not quite as long as body, relatively slender; scape subcylindrical beyond basal third, finely punctured; third segment about as long as scape; fourth fourfifths as long as third; fourth to tenth gradually shorter; last as fong as sixth, attenuated and acuminate apically. Prothorax broader than long, constricted near apex and between middle and base, swollen on center of disc, and coarsely punctured. Soutelulm short and broad, subtruncate posteriorly. Elytra fairly long, silghtly broader near apices than at bases, each impressed with about twelve subregular longitudinal rows of moderate punctures. Ventral surfaces sparsely punctured at sides. Legs fairly slender. Length 10.7 mm.; breadth 3.6 mm.

Holoytpe, female (Lingnan Natural History Museum), Tai-pin-ts'uen (大邊村) alt. 400 meters, Lam-ka-heung, Lai-mo-ling (Loi Mother Mt.), Kiung-shan Distr., central Hainan (海南島), May 14, 1935, F. K. To.

Differs from *A. parva* Gressitt (1935, Philippine Journ. Sci., 57: 193, Formosa) in being larger, with the frons and vertex more heavily punctured, the pronotum more swollen and much more strongly punctured, particularly on anterior portion, and the elytra a little less regularly punctured and a little less strongly so behind middle.

Distribution.—Hainan Island.

18. *ANASTATHES ROBUSTA* Gressitt.

Anastathes robusta Gressitt, 1940, Philippine Journ. Sci., 72: 222 (Hainan).

Body ochraceous. reddish on head and pronotum, slightly so on abdomen; elytra pale orange testaceous; antennae black. Dorsal surfaces with some erect goldish hairs, but almost lacking pubescence. Pronotum with a very large swelling, reaching almost to apex and base, heavily punctured; elytra with moderate punctures in subregular longitudinal rows on basal two-thirds. Length 9.5-11.5 mm.; breadth 3.6-4.6 mm.

Distribution.—Hainan Island.

Genus *ASTATHES* Newman

Astathes Newman, 1842, Entomologist, 1: 299; Thomson, 1860, Classif. Ceramb.: 42; 1864, Syst. Ceramb.: 399; Pascoe, 1867, Trans. Ent. Soc. London, (3) 3: 348; Lacordaire, 1872, Gen. Col., 9: 872, 873; Gahan, 1901, Trans. Ent. Soc. London: 38; Gressitt, 1940, Philippine Journ. Sci., 72: 220.

Tetraophthalmus Guerin, 1844, Icon. Regne Anim. Ins.: 244; Thomson, 1857, Archives Ent., 1: 48.

Tetraophthalme Blanchard, 1845, Nat. Ins., 2: 161.

Body broad, oblong; head as wide as prothorax; front of head convex; prothorax broader than long, swollen on center of disc and at middle of each side; elytra broadly rounded apically; intercoxal process of mesosternum nearly vertical anteriorly, over-lapped posteriorly by a long anterior process of metasternum, which reaches almost to anterior borders of middle coxae.

Genotype.—*Astathes perplexa* Newman.

Range.—Oriental Region; Wallacea.

KEY TO THE CHINESE SPECIES OF ASTATHES

1. Antennae largely black 2
   Antennae yellow .... *flavicornis*

2. Elyta greenish or steely blue; third to fifth antennal segments largely ochraceous .... *cyanoptera*
   Elytra purplish blue; third antennal segment black, fourth to sixth black distally .... *episcopalis*

19. *ASTATHES CYANOPTERA* Gahan.

Astathes cyanoptera Gahan, 1900, Ann. Mag. Nat. Hist., (7) 5: 353 (Hainan Island); 1901, Trans. Ent. Soc. London: 44; Matsushita, 1933, Journ. Fac. Agr. Hokkaido Imp. Univ., 34: 430; Gressitt, 1940, Philippine Journ. Sci., 72: 221, pl. 6, fig. 14.

Reddish ochraceous; elytra greenish or greenish blue; antennae pitchy black on scape and sixth to last segments; tarsi and apices of tibiae blackish. Pronotum strongly swollen, sparsely and finely punctured on swelling; elytra strongly and irregularly punctured. Length 11.5-13 mm.

Distribution.—Hainan Island; Formosa.

20. *ASTATHES EPISCOPALIS* Chevrolat.

Astathes episcopalis Chevrolat, 1852, Revue Zool., (2) 4: 418; Gahan, 1901, Trans. Ent. Soc. London: 44; Savio, 1929, Notes d'Ent. Chinoise, 1 (4): 3; Matsushita, 1933, Journ. Fac. Agr. Hokkaido Imp. Univ., 34: 430; Liu, 1934, Lingnan Sci. Journ., 13: 660; Pic, 1935, Arkiv f. Zool., 27 A 2: 12; Wu, 1937, Cat. Ins. Sin., 3: 775; Gressitt, 1939, Lingnan Sci. Journ., 18: 110; 1940, Notes d'Ent. Chinoise, 7: 197.

Tetraophthalmus episcopalis Thomson, 1857, Archives Ent., 1: 48.

Reddish ochraceous; elytra purplish blue; antennae black with most of fourth segment, and bases of fifth to seventh, pale ochraceous; tibiae and tarsi black. Pronotum moderately swollen in center, with coarse punctures on sides of swellings; elytra with fairly close and deep punctures, becoming smaller and sparser at apices. Length 10.5-16.3 mm.

Vol.XI 第十二巻B 1940 3361

One specimen was collected at Tau-chi-tsz (桃溪寺), near Tsengyih (遵義), alt. 250 meters, northern Kweichow Province (貴州), July 14, 1940, by the writer. New to Kweichow. Some specimens were taken at Ta-o-sze (大峨寺), Omei Shan (峨眉山), Szechuan, in July 1940, by Dr. C. C. Liu; specimens in Univ. Nanking Coll. Agr. from Chengtu (成都) and Kuanhsien (灌縣). The Academia Sincia has specimens from Chekiang.

Distribution.—Shensi, Szechuan; Kweichow; Anhwei; Kiangsu; Chekiang; Kiangsi; Fukien; Kwangtung; Kwangsi; Hong Kong; Formosa.

21. *ASTATHES FLAVICORNIS* Pic.

Astathes flavicornis Pic, 1939, Echange, 55: 31 (China).
Distribution.—China.

EXPLANATION OF PLATE

(Magnified 6.8 times) (allowing ¾ reduction)

Figure 1. *Chreonoma rigida* Gressitt, new species, holotype.
Figure 2. *Chreonoma dioica* (Fairmaire); Chengtu.
Figure 3. *Anastathes parva hainana* Gressitt, new subspecies, holotype.
Figure 4. *Plaxomicrus ellipticus* Thompson; Chengtu.

華西邊疆研究學會雜誌
3362

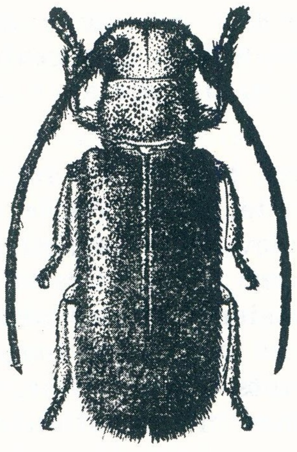

1

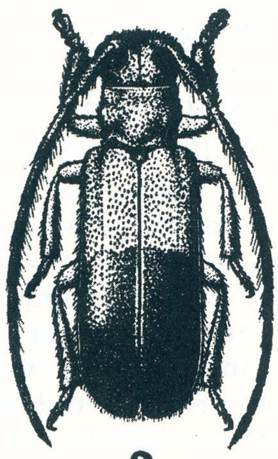

2

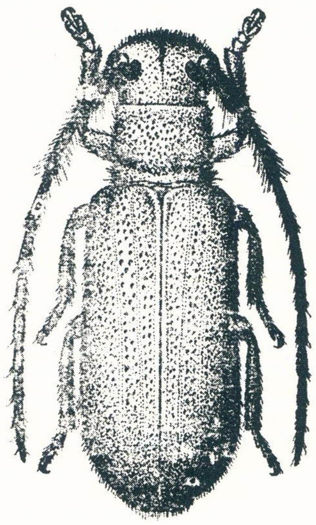

3

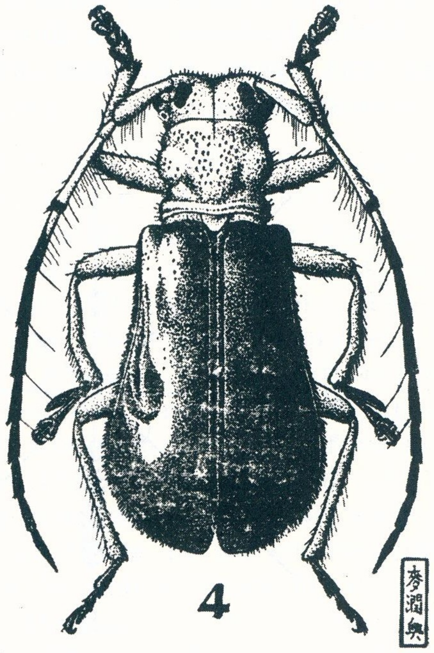

4

麦潤奥
